# Comparing the stability of implants coated with nano PLGA-alendronate sodium: an in-vivo study

**DOI:** 10.2340/biid.v12.43372

**Published:** 2025-03-31

**Authors:** Vahid Naemi, Abbas Haghighat, Mahla Esfahanian

**Affiliations:** a Department of Oral and Maxillofacial, Faculty of Dentistry, Golestan University of Medical Sciences, Gorgan, Iran; b Torabinezhad Research Center, Faculty of Dentistry, Isfahan University of Medical Science, Isfahan, Iran; c Dental Research Center, Faculty of Dentistry, Golestan University of Medical Sciences, Gorgan, Iran

**Keywords:** PLGA-alendronate, implant anchorage, dip coating

## Abstract

**Objective:**

Considering the effect of bone resorption-inhibiting drugs, such as bisphosphonates, on bone density, the present study evaluated the stability of implants coated with polylactic-co-glycolic acid (PLGA)-alendronate sodium.

**Methodology:**

Ten healthy Swiss rabbits were selected (mean weight: 3.5 kg). Two titanium implants were inserted in each rabbit’s tibia bone, one implant coated with PLGA-alendronate sodium and the other functioning as control. Torque meter equipment measured the amount of torque while inserting the implants. Insertion torque was measured during the initial insertion of the implants, and after 4 weeks, the rabbits were anesthetized again, the implants were exposed, and resistance torques were measured and recorded. Using a trephine bur, all implants and the surrounding bone structure were then removed for histological evaluation. Fourier transform infrared spectroscopy was used to investigate alendronate and PLGA biologically. Data analysis was performed using SPSS.v23 software with a significance level of *p* < 0.05.

**Results:**

The results showed that the difference between insertion torque and final resistance torque after 4 weeks was statistically significant (*p* = 0.024). The mean number of osteoclasts and macrophages differed substantially between the experimental and control groups (*p* < 0.001). The ratio of woven bone to lamellar bone and the number of osteoblasts did not differ significantly between the two groups (*p* > 0.05).

**Conclusion:**

The use of PLGA-alendronate sodium coating decreased the number of osteoclasts and macrophages and increased the stability of implant anchorage.

## Introduction

The surface of implants can be coated with bone-stimulating substances, such as growth factors, to accelerate the healing process locally [1]. The limiting factors of this method are that the active ingredients are released gradually, and there is no single initial dose. These surfaces can also be loaded with molecules that control bone remodeling [2]. Combining bone resorption inhibitors, such as bisphosphonates, can be helpful in cases where bone-supporting tissue is lacking. It has been shown that loading bisphosphonate on an implant leads to increased bone density in the area around it. On the other hand, in some cases, surgical procedures such as ridge augmentation, sinus augmentation, or bone grafting are necessary before implantation to improve the quality and quantity of the recipient bone [3]. For some patients, implanting is impossible without increasing the vertical or horizontal ridge [4]. Therefore, improving the quality of bone with the help of ossification-stimulating drugs will reduce the need for such surgeries.

A new method for improving the surface properties of all types of intraosseous implants is surface coating with proven effective biomaterials. The polymer can be degraded by polylactic-co-glycolic acid (PLGA) and can improve function at the bone contact surface as a local drug carrier system. Drugs mixed into polymers are released continuously over several weeks and mitigate the consequences [5, 6]. The advantages of this method include local and controlled release of materials in combination with polymer, gradual absorption of lactate and its complete metabolism in the body, and increasing the stability of implants in combination with a drug delivery system [7, 8]. Topical application of alendronate has been shown to reduce bone resorption, which routinely occurs in dental practices where the periosteum is wholly separated from bone [9]. Therefore, a topical delivery system with excellent pharmaceutical performance is required.

The current study aimed to compare the strength around implant anchorage (bone implants) coated with nano PLGA-alendronate sodium and suitable conditions designed to optimize the loading and storage of alendronate in PLGA micro and nanosphere particles using the W/O/W technique with the help of sodium. The optimized formula on the coated implants and the effectiveness of the surface of the titanium implants in in-vivo conditions were evaluated in terms of strength, and the histomorphometrics of the tissue around the implant anchorage were assessed. The current in-vivo study examined the clinical and histological stability of bone implants (implant anchorage) coated with PLGA and sodium alendronate compared to the control group. The hypothesis was that implants coated with nano PLGA-alendronate sodium would increase the stability of bone screws, change the histological structure of the tissue around the implants, and reduce the number of osteoclasts and macrophages.

## Materials and method

### Animals and experimental design

The present study was conducted with ethical code IR. ISUMS.REC. 391363 from the Isfahan University of Medical Sciences. Ten healthy, 9-month-old, Swiss rabbits of both sexes and a mean weight of 3.5 kg were randomly selected. Following anesthesia with ketamine and xylazine at doses of 35 and 5 mg/kg, each rabbit received two titanium implants, an implant covered with nano PLGA-alendronate sodium in one leg bone (tibia) and a control implant in the other leg bone.

The 6 mm implants with a diameter of 1.6 mm were inserted according to the manufacturer’s recommendations (Jeil, Seoul, South Korea). The operations were performed under normal cooling conditions and at low speed (2 rps) to prevent thermal trauma to the bone. The implants used were self-tapping implants and to prevent bone fracture due to pressure, the entry point in the cortex was pierced with a bur. At the time of implant insertion, the amount of torque generated was recorded with a torque meter model DID-4 (Cedar, Ibaraki, Japan) , the soft tissue was closed with simple absorbable sutures, and the skin was closed with simple sutures with 0–3 nylon suture. After surgery, animals received enrofloxacin (10 mg/kg) subcutaneously daily for 5 days, and Flunixin meglumine (1.1 mg/kg) was also prescribed for pain relief.

Insertion torque was measured during the initial insertion of the implants, and after 4 weeks, the rabbits were anesthetized again, the implants were exposed, and resistance torques were measured and recorded. For mechanical analysis, torque was measured continuously during the insertion and removal of implants, and maximum torque was extracted from these measures. Total insertion energy was calculated during placement to the maximum torque point. Total removal energy was calculated from the maximum torque point to complete removal.

Then, the implants along with the surrounding bone along their longitudinal axis were removed by a dental trephine bur for histological examination. The rabbits were sacrificed using an overdose of anesthetic.

### Preparation of nanospheres

In the present study, PLGA nanospheres were prepared by the W/O/W double emulsion method, in which PLGA polymer was dissolved in polyethylene glycol (PEG) [10].

In agreement with the W/O/W double emulsion method, 7 mg of sodium alendronate was added to 170 mg of water and dissolved in it, forming the first aqueous phase W1. The organic phase was formed by dissolving 200 mg of PLGA in 3 mL methylene chloride. The emulsion was homogenized with a high-pressure homogenizer for 2 minutes and added to 250 mL of 1% Polyvinyl Alcohol (PVA) solution. The emulsion stabilizer was placed in an ultrasonicate. The other aqueous phase containing NaCl at a concentration of 1% was mixed at a speed of 1,500 rpm with a magnetic stirrer to form a W/O/W emulsion. Stirring was continued for 4 hours to release and evaporate the PEG and harden the nanospheres. The polymer particles were washed in distilled water and dried under vacuum at 37°C for 24 hours to evaporate the solvent and obtain the PLGA/AL microspheres.

### Coating implants with PLGA and alendronate

The prepared W/O/W emulsion was then cooled in an ice bath to increase its viscosity and reduce evaporation after application. The coating process was carried out by immersing the sterile anchor implants in the emulsion using the dip coating process; the coated implants were then placed in specific NaOH (Sodium Hydroxide concentrations (n10–0.1) at different times from 1 to 60 minutes. Then they were washed in non-ionized water until they reached a constant pH of 7. Finally, the implants were sterilized by exposing them to Ultraviolet (UV) light for 2 hours and then soaking them in 70% ethanol overnight. With this process, the implant obtained a surface in the form of a nanostructure with a particle size of approximately 200–300 nm.

### Preparation of histology samples

The bone samples containing the implant removed from the rabbit tibia were fixed with 10% formalin (FBS) for 2 days and then with 10% EDTA (Ethylenediaminetetraacetic Acid) in 0.1 L/mol hydrogen chloride (HCl)-Tris buffer solution at 4°C for 4 days. Subsequently, the decalcified samples were embedded in paraffin, and microtomes with a diameter of 4 μm were cut and stained with hematoxylin and eosin. A microscope with 10× magnification equipped with a digital camera was used to photograph the samples. The sampling was done with a risk of α 0.05 and a power of 80%, and with a probability of 80%, and a difference of 20.7 Nm in lSQ was observed when selecting 10 implants in each group (control and experimental).


$$n=\frac{{\left(z_{1-\frac{\alpha }{2}}+z_{1-\beta }\right)}^{2}(\delta_{1}^{2}+\delta_{2}^{2})}{d^{2}}$$

$$\alpha =0.05\to Z_{1\frac{\alpha }{2}}=1.96$$

$$1\;–\;\beta \;=\;0.80\to Z_{1–\beta }\;=\;0.84$$

δ: R6 = 996 = 16.5

R: 100-1 = 99

D = 20/66 = 20/7

n = 10

### Fourier transform infrared analyses

Fourier transform infrared (FTIR) spectra for PLGA and PLGA/AL microspheres were recorded on an Avatar Nicolet spectrophotometer in KBr pellets, within the range 400–3,000 cm -1. The transmittance and wavelength were measured separately and combined.

### Histological image

The alterations of macrophages and osteoblasts were evaluated using histology and immunohistochemistry techniques. Under light microscopy (Olympus CX21FS, Olympus Corporation, Tokyo, Japan) with magnifications of 40× and 400×, the number of osteoclasts in the bone adjacent to the implant with a radius of 1 mm was counted. Slides were evaluated for type of bone. The total percentage of bone around the implant, and the woven bone, lamellar bone and mentioned radius were calculated by Iranian histomorphometric analysis (HMMA) ver. 1 [11].

### Data analysis

Data analysis was performed using SPSS.v23 software with a significance level of *p* < 0.05. An independent *t*-test was used to compare the two groups.

## Results

The results of the torque measurements are presented in Tables 1 and 2. The mean difference between insertion torque and final resistance torque was 1.6875 for the experimental group (*n* = 10) and -0.1500 for the control group (*n* = 10) (Table 1). The strength around the implants in the experimental group was higher than the control group (*p* < 0.001).

**Table 1:** Statistical index of mean torque in control and experimental group.

Group	Number	Mean torque difference	SE	*P*
Experimental	10	1.6875	0.58310	0.0001
Control	10	–0.1500	0.38658	

**Table 2:** The mean difference of insertion torque and final resistance torque before and after 4 weeks.

Variable		Experimental	Control	*P*
Insertion torque	Before	–0.773	1.529	-
	After	–4.365	0.257	
*P*		0.003	0.804
Final resistance torque		–2.865	–0.321	0.024

Insertion torque and final resistance torque were significantly higher in the experimental group than in the control group (*p* < 0.05) (Table 2).

**Figure 1:**
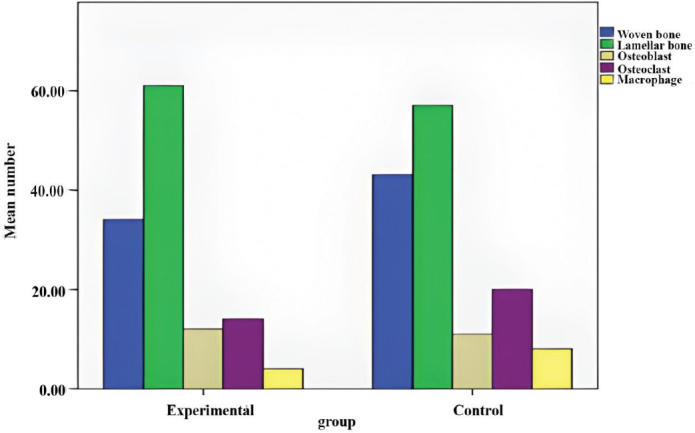
Mean number of cells (osteoblast, osteoclast and macrophage) and mean percentage of bone (woven bone and lamellar bone) in the experimental and control groups.

The histological results are presented in Figure 1 and Table 3. There was a statistically significant difference in the mean number of osteoclasts (experimental: 18; control: 20) and macrophages (experimental: 2; control: 8) between the experimental and control groups (*p* < 0.01).

**Table 3:** Mean difference in cells numbers and mean percentage of bone in the experimental and control groups.

Variable	Experimental	Control	*P*
Woven bone	0.342	–2.152	0.060
Lamellar bone	11.805	1.843	0.098
Osteoblast	2.867	1.408	0.193
Osteoclast	–3.099	–4.679	< 0.001
Macrophage	–0.039	–2.530	0.035

However, the ratio of woven bone (experimental: 38; control: 43), lamellar bone (experimental: 61; control: 57), and the number of osteoblasts (experimental: 12; control: 11) did not differ significantly between the two groups (*p* > 0.05).

**Figure 2:**
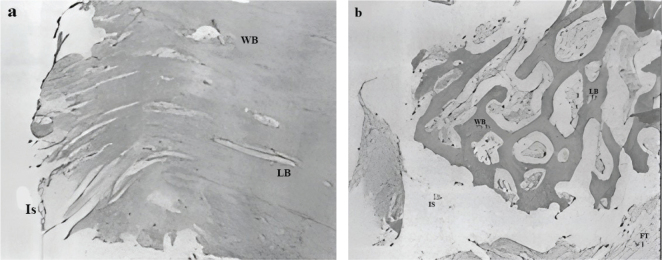
Histological image prepared under light microscope (H&E staining), X40 magnification of control group (a) and experimental group (b). WB: woven bone; LB: Lamellar bone; FT: Fibrotic tissue; Is: Implant surface.

Representative histological images of the experimental and control samples are shown in Figure 2. Differences in the total number of osteoclasts and in the ossification pattern between the two groups are obvious. Lamellar bone is also evident in the experimental group. Statistical index of mean osteoblast, osteoclast, macrophage, lamellar bone and woven bone are given in Table 4.

**Table 4:** Statistical index of mean osteoblast, osteoclast, macrophage and lamellar and woven bone.

Group	Mean and Std. deviation	Woven bone	Lamellar bone	Osteoblast	Osteoclast	Macrophage	Dif
Experimental	Mean	36.20	62.40	11.60	13.80	3.67	–0.15
	N	10	10	10	10	9	8
	SD	7.391	7.043	1.897	3.967	0.707	1.649
Control	Mean	42.90	57.10	10.50	19.80	4.10	1.69
	N	10	10	10	10	10	8
	SD	3.604	3.604	2.014	2.440	0.738	1.093

**Figure 3:**
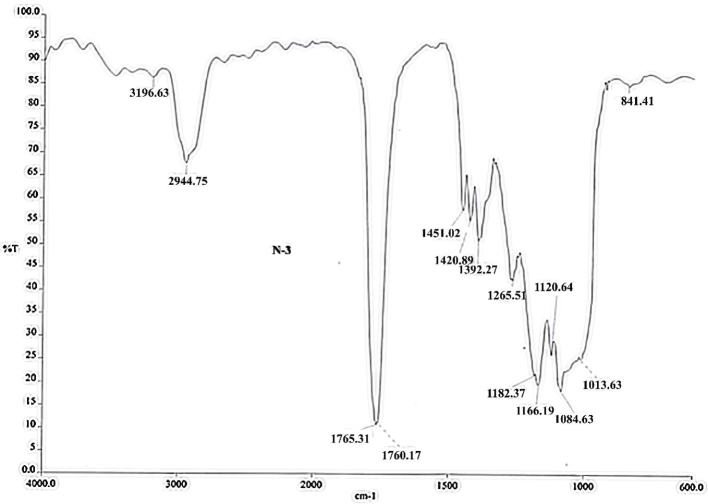
FTIR diagram of PLGA-alendronate sodium. FTIR: Fourier transform infrared; PLGA: polylactic-co-glycolic acid.

The FTIR spectra for PLGA and PLGA/AL microspheres are shown in Figure 3. The spectra were recorded separately, first for alendronate sodium and then for PLGA. Finally, the FTIR spectrum of the final material was measured, which was a mixture of PLGA and alendronate. The presence of wave peaks of alendronate sodium (at 1,013 cm^-1^) and PLGA (1,760 cm^-1^) in the final material indicates the successful mixing of the two. The FTIR diagram shows a successful combination of two materials and the coating process.

## Discussion

In the present study, alendronate was used as a second-generation bisphosphonate (BP) to reduce osteoclast activity and, as a result, to increase initial implant stability. The effect of local and systemic use of alendronate has been studied in numerous articles on the osseointegration of dental implants [12] and the treatment of defects around the implant [13, 14]. However, few studies have been conducted on the effects of its use in intraosseous implants. In the present study, alendronate coating of implants was used in topical form. The advantage of using topical form compared to systemic form is that there is no injury at the injection site, a higher dose of the drug can be prescribed near living bone, or there is no concern about poor gastrointestinal absorption [15].

In the present study, all guidelines for the care, maintenance and treatment of animals were followed. The use of PLGA and alendronate also seems to have no risk for animals, considering the many uses in past studies [16]. Rabbit spongy bone was chosen for this study because it has similarities with human bone [17]. Ten healthy Swiss rabbits survived the anesthesia and the surgical procedure without complications. All implants could be inserted into the tibia bone without any particular problems. None of the rabbits had a hematoma, infection or any particular complications.

PLGA is a good candidate for a delivery system due to its good adhesion to cells [18]. Another desirable feature being considered for PLGA is the ability for controlled drug release [19]. This release can vary between 48 hours and 3 months depending on the composition and molecular weight, the substances added, and the conditions under which the substance was produced. Considering the above features, PLGA was used as delivery system for alendronate in the present study. Studies suggest the use of multiple emulsion techniques as an alternative to single emulsion techniques [20]. The reason for this choice is the possibility of encapsulating hydrophilic drugs in polymeric microspheres to increase effective loading. In the case of alendronate, it was shown that the use of a 0/w mono-emulsion technique results in poor entrapment in PLGA microspheres [21]. Therefore, the W/O/W multiple emulsion technique was used in the present study.

Meraw et al. reported that alendronate increases the early bone formation rate around dental implants [22]; this result is consistent with that of the present study, although the difference between the present study and the above study was the use of anchorage implants instead of dental implants. Kajiwara et al. showed a positive effect after 4 weeks of bone implants impregnated with BP compared to titanium implants without covering material [12]. This study agrees with our study regarding examining the bone implant in the tibial bone and the positive result of BPs. However, in the said study, pamidronate was used instead of alendronate, and bone formation was also examined using images obtained using confocal laser scanning microscopy.

In the present study, torque findings showed that insertion torque was significantly higher than final resistance torque in experimental group, while the histological findings showed the decrease in the number of osteoclasts and macrophages.

The histological evaluation also found that the number of osteoblasts in the experimental group increased compared to the control group, although this difference was not statistically significant. According to a study conducted by Gun et al., BP can have a mitogenic and stimulating effect on osteoblasts [23]; also, BPs inhibit osteoblast apoptosis [24–26]. In the present study, alendronate led to a significant decrease in the number of osteoclasts in the experimental group compared to the control group. Rogers et al. (1996) reported that the use of BPs can cause changes in the morphology of macro cells (MAC) cells and nuclei, and finally MAC apoptosis [27]. Vertesich et al. used a physiologically loaded mouse implant model to investigate the short-term effects of postoperative systemic alendronate on osseointegration, and showed that alendronate decreased peri-implant osteoclasts while preserving peri-implant osteoblasts and endothelial cells [28]. In the present study, the ratio of woven bone to lamellar bone in the control group was higher than in the experimental group, although this difference was not statistically significant, but as already mentioned in the studies, BP causes a decrease in osteoclasts, which leads to a reduction in the bone turnover and increased osteoblast activity. Therefore, it is logical that the ratio of lamellar bone to woven bone is higher in the experimental group. Jolic et al. in a review study evaluated the impact of medication on osseointegration and implant anchorage in bone determined using removal torque, and showed while morphometric parameters such as bone-implant contact appear to influence the biomechanical anchorage, increased or decreased corresponding fluctuations in bone-implant contact do not always accompany removal torque [29].

## Conclusion

According to the present results, using PLGA-alendronate sodium coating increased the stability of implant anchorage. Using these coated implants caused a change in the histological structure of the tissue around the implants in the form of a significant reduction in the number of osteoclasts and macrophages. Since intraosseous implants used for osteosynthesis are exposed to early forces, it may be possible to give them more stability by utilizing an alendronate coating.
